# How can the ethical conduct of verbal autopsies be enhanced? Lessons from Southeast Asia

**DOI:** 10.1080/11287462.2025.2550810

**Published:** 2025-09-02

**Authors:** Nan Shwe Nwe Htun, Bipin Adhikari, Aung Pyae Phyo, Carlo Perrone, Koukeo Phommasone, Nawrin Kabir, Moul Vanna, Yoel Lubell, Thomas J. Peto

**Affiliations:** a Faculty of Tropical Medicine, Mahidol Oxford Tropical Medicine Research Unit, Mahidol University, Bangkok, Thailand; b Nuffield Department of Clinical Medicine, Centre for Tropical Medicine and Global Health, University of Oxford, Oxford, UK; c Faculty of Tropical Medicine, Shoklo Malaria Research Unit (SMRU), Mahidol-Oxford Tropical Medicine Research Unit, Mahidol University, Mae Sot, Thailand; d Lao-Oxford-Mahosot Hospital Wellcome Trust Research Unit, Mahosot Hospital, Vientiane, Lao People's Democratic Republic; e Communicable Diseases Programme, BRAC, Dhaka, Bangladesh; f Action for Health Development, Battambang, Cambodia

**Keywords:** Death, bereavement, ethics, verbal autopsy, Southeast Asia

## Abstract

Verbal autopsy research is vital for understanding community mortality, informing health interventions and policies in low- and middle-income countries. However, overlooking the community perspectives on deaths can undermine the ethical conduct and effectiveness of such research. This study explored community-based concepts of death, interpretations, and coping mechanisms in five Southeast Asian countries, with this manuscript highlighting key findings from the body mapping exercise that revealed diverse cultural and religious understandings on death. Participants’ views ranged from seeing death as a cessation to life's struggles to an inevitable end, reflecting deep cultural and spiritual beliefs. Coping mechanisms, often grounded in religious practice and community support, played a crucial role in managing grief. The study also underscores the importance of addressing emotional well-being for both participants and researchers. Recommendations include integrating mental health support into research protocols and tailoring practices to local cultural contexts. These findings inform the design of more ethically grounded verbal autopsy tools and procedures that are sensitive to local beliefs and emotional dynamics, ultimately improving data quality and community trust.

## Introduction

Verbal autopsy (VA) is widely used to identify causes of death in low and middle-income countries (LMICs), where deaths often occur at home and the functional vital registration system is largely absent. VA collects vital information on symptoms and events leading to death through interviews with the deceased person's next of kin or caregivers (WHO, 2022). Nonetheless, the sensitive nature of VA can cause emotional distress for both participants and interviewers, potentially affecting the data quality (Hinga et al., 2021). VA has been increasingly employed across Southeast Asia (SEA) and other LMICs to fill gaps in mortality data; however, standardized ethical guidelines to safeguard the emotional well-being of all parties involved remain limited (Byass & Chandramohan, 2020; Hinga et al., 2021).

The SEA region comprises countries with rich cultural, religious, and linguistic diversity, which presents both opportunities and challenges in conducting VA research. Concepts of death in SEA are deeply rooted in cultural, religious, and spiritual beliefs (Wessing, 2004). Traditions such as Buddhism, Islam, Christianity, Hinduism, and various indigenous animist practices shape these perceptions, framing death through specific spiritual and cultural lenses as a release from suffering, a journey to the afterlife, or a continuation of karmic cycles (Cann, 2014; Cohen, 2008). These culturally grounded beliefs influence the VA research process, from how families grieve, to their willingness to participate, to their comfort in discussing the deceased, to the interpretation of pre-death symptoms and events. In recent years, countries such as Myanmar, Philippines and Vietnam (Boyce & Reyes, 2020; Hazard et al., 2020) have considered incorporating VA into their routine civil registration and vital statistics systems; however, this expansion raises important ethical and social considerations, particularly in the multicultural and socioeconomically marginalized settings. To date, the ethical concerns around VA in SEA settings have not been thoroughly reported although they are relevant to global bioethics research principles in general. Exploring ethical principles in VA research including respecting the autonomy and dignity of the deceased and their families, protecting participants from harm, acknowledging cultural sensitivities, and ensuring equitable inclusion of diverse and often underserved populations can enhance the effectiveness of such studies. It can also improve communities’ acceptance.

As part of the South and Southeast Asian rural febrile network research program (Chandna et al., 2021), the multi-country VA study was conducted across five countries with the goal of strengthening regional mortality data. Prior to data collection, public engagement activities incorporating qualitative approaches were undertaken, recognizing their important role in community-based research. These efforts helped foster trust, deepen understanding of local contexts, and promote active participation (Jacquez, 2020). In particular, a participatory body mapping exercise was employed to explore community members’ understanding of death including symbolic interpretations, emotional responses, and coping mechanisms. This manuscript presents key insights from that exercise, which contribute to the ethical discourse on implementing VA in culturally diverse, resource-limited settings.

## Materials and methods

### Study sites and cultural context

The public engagement activity took place in selected rural areas of SEA that included Bangladesh, Thai-Myanmar border, northern Thailand, southern Lao PDR, and western Cambodia between October 2021 to March 2023. These sites were selected to reflect a wide range of cultural and religious practices surrounding death and bereavement.

Northern Thailand: Predominantly Theravada Buddhist, where death is viewed as part of the rebirth cycle (samsara), and merit-making rituals such as food offerings to monks and temple ceremonies are central to mourning (Cohen, 2008).

Thai-Myanmar Border: Home to ethnically diverse communities, including Karen, and Mon communities, with spiritual practices rooted in Buddhism, Christianity, and animism. Beliefs in ancestral spirits and lingering souls shape local death customs (Antoni, 1982; Endres & Lauser, 2012).

Southern Lao PDR (Savannakhet province): A region, where Theravada Buddhism blends with indigenous animist traditions. Common rituals include cremation, chanting by monks, and offerings to support the soul’s transition (Antoni, 1982).

Western Cambodia (Pailin and Battambang Provinces): Strongly influenced by Buddhist and ancestor worship. Death anniversaries, communal pagoda ceremonies and public commemorations are widely practiced (Cohen, 2008; Endres & Lauser, 2012).

Rural Bangladesh (Cox’s Bazar and Bandarban districts): Predominantly Muslim, where death is viewed as divine will. Burial and immediate mourning rituals such as prayers and Qur’an recitation are practiced, with variations across communities (Rashid, 2020).

### Methods

The public engagement involved two main components: focus group discussions (FGDs) and a participatory body mapping exercise. Experienced local researchers, using the local language and adhering to standard body mapping and FGD guidelines led the activities at the study sites (Boydell, 2020). Eight to twelve participants formed a group across all sites (a total of seventy individuals), were purposively selected to ensure demographic diversity. Detailed participants characteristics and selection criteria were described elsewhere (Htun et al., 2023). FGD began with open-ended questions about customs and perceptions of death before gradually moving to specific questions about the VA study. Following this, participants engaged in a body mapping exercise, a participatory arts-based research technique that uses visual representation to explore individuals’ experiences, emotions, and beliefs (Boydell, 2020). In our study, participants first created symbolic representations of their perceptions of death on A4 paper and then translated these into life-sized body maps while engaging in discussion with facilitators. To assist interpretation, color psychology (Alnasuan, 2016) was also used as a guide for interpreting emotions and behaviors, for example, red and black symbolized grief, loss, pain and mourning while green and blue represented healing, nature and spirituality. These visual expressions helped us uncover deeper layers of emotional and cultural meaning that participants might not have articulated verbally. This method was particularly valuable in culturally diverse where conventional verbal methods may be limiting due to language or literacy barriers. Verbal and visual data from both methods were collected, transcribed, and thematically coded. However, this manuscript focuses exclusively on the findings from the body mapping activity. The results of the FGDs were already discussed in a separate publication (Htun et al., 2023). The body mapping exercise provided a foundational understanding of local perceptions of death and informed culturally appropriate and ethically sensitive adaptations to the VA process.

While participants were drawn from diverse cultural settings, the analysis aimed to identify overarching themes relevant to the ethical implementation of VA across the region. Because of the limited number of participants per site and the exploratory nature of the body mapping activity, this manuscript does not provide a comparative analysis across the sites. Instead, we focused on cross-cutting themes that emerged consistently across all settings.

## Ethical approval and consent

Ethical approval for this study was obtained from the National Ethics Committee for Health Research, in Cambodia and Lao PDR; BRAC James P Grant School of Public Health, Bangladesh; the Karen Department of Health and Welfare for the Thai-Myanmar border; Chiang Rai Provincial Public Health Office, Northern Thailand; and the Oxford Tropical Research Ethics Committee, UK (OxTREC 553-20). Written informed consent was obtained from all participants prior to participation, with the process conducted in the local language by trained researchers. Participants were informed of their rights, including the voluntary nature of participation and the option to withdraw at any point. Confidentiality and cultural sensitivities were rigorously respected throughout the study.

## Results

The two main themes identified from the study were: (1) conceptualization of death, and (2) expression of grief and coping mechanisms.

### Conceptualization of death

In rural SEA communities death was perceived as an inevitable part of life, yet its meaning was shaped by distinct spiritual and cultural perspectives, viewed variously as a release from worldly suffering, a passage to the afterlife, or a continuation of karmic rebirth. This highlights the need to understand local interpretations rather than assume a universal response. Death held deep social and cultural significance, often framed by religious teachings and community norms. Notable remarks included a Muslim participant who said, “*Surely we belong to Allah, and to Him we shall return,”* while Buddhist participants shared, “*All conditioned things are impermanent; all conditioned things are painful, and all dhammas are without self.*”

Among the ethnic minorities living in Thailand, such as the Lisu, Lahu, and Akha – who are predominantly Christian – there is a shared belief that one's actions leave a lasting legacy: “*Live your life with purpose before you die. Work hard, avoid laziness, and don't follow the bad examples set by others. Focus on doing good things.”* Another added, “*At the end of our lives, only the good and bad deeds we have done will remain with us.”*

Thai communities often perceived death as a guiding star or a beacon that led them forward. One noted, “*When a person dies, I think of a candle; it’s like there is a light leading the way.”* In contrast, a Cambodian mother who lost her younger son touched on a common belief, yet expressed profound personal grief “*People say good deeds can influence longevity, but the loss of my son still fills me with overwhelming grief, bringing tears and leaving me speechless. Despite my efforts to stay calm, the pain remains ever-present.”* A Karen participant living on the Thai-Myanmar border expressed a different view: “*Death is a liberation from the struggles of life.”* These diverse perspectives highlight the varying ways death is perceived across Asian cultures, often blending spiritual, philosophical, and cultural elements.

Many participants viewed death as unpredictable, causing anxiety and fear about the abrupt end of their responsibilities, desires, and ambitions. Some admitted feeling anxious, scared, and uncomfortable when contemplating their own mortality. Understanding and respecting these local customs became crucial for researchers during the data collection and was observed through progressive development of candid conversations, sharing concepts, interpretations and finally their coping mechanisms.

### Expression of grief and coping mechanisms

Expression of grief and coping mechanisms exhibited both diverse and notable similarities. These responses were influenced by religion, societal norms, and personal beliefs. For many, religion often served as a major source of comfort, helping alleviate the sorrow and despair of losing close family members and friends. Common visual symbols used to express grief included broken hearts and teary-eyed to represent sadness, prayer hands to signify hope and spirituality, and candles used for remembrance.

Spiritual and religious coping practices were widespread. Participants described visiting sacred places such as pagodas, temples, churches, or mosques and seeking guidance from monks or religious leaders, depending on their faith and cultural background. One participant illustrated a monk and a temple, stating, “*When I feel overwhelmed by grief, I visit temples and seek comfort and guidance from monks or religious leaders*.” Such rituals helped individuals release from the emotional attachment, move forward after a loss and return to daily routines, a process often supported by societal expectations of resilience.

Support from family and friends also played a critical role in coping. One participant shared, “*Just being around family and friends made the pain easier to bear*,” highlighting the importance of social support in alleviating emotional distress. Others described coping strategies involving physical and emotional distancing from their usual environment. One participant remarked, “*Traveling helped me get out of my usual space and stop thinking about the loss all the time*.” Nature emerged as another important source of comfort. A participant who drew a forest and mountain explained, “*Being in nature gave me peace and helped me clear my mind.*” Additionally, some respondents found solace in spiritual or auditory distractions such as listening to religious teachings or music. As one individual noted, “*I try to listen to Dhamma talks or music to distract myself from grief*.” These visual and narratives accounts reflect the diverse ways individuals seek comfort and psychological relief following bereavement. Moreover, in our study communities, bereavement was marked by large religious gatherings where food and alcohol were served, providing space for shared mourning and practical support. During such gatherings, attendees often expressed their sympathy to the bereaved family, offering material, financial, and spiritual support, strengthening social cohesion and reassuring families they would not be left alone.

Along these conceptualizations of deaths and coping mechanisms, interviewers and researchers reflected on the challenges and opportunities for improving VA research, particularly drawing on the implications for ethical conduct and greater acceptability within the communities. Although participants’ cultural and emotional insights were not always explicitly related to VA, they provided valuable guidance for enhancing the ethical sensitivity, cultural appropriateness, and overall conduct of VA procedures. Such adaptations are crucial for building trust, reducing emotional burden, and increasing community acceptance of the VA process. In the following discussion, we reflect on the findings identified in this study align with global bioethics principles and consider their implications in relation to the broader literature.

## Discussion

The study highlights the cultural beliefs, religious practices, and the perception of death in SEA, with particular emphasis on how these elements influence VA research. The findings reinforce the need for culturally responsive approaches, especially in a region as diverse as SEA, where ethnic, religious, and cultural practices significantly shape individuals’ experiences, interpretations and coping with death.

Our findings highlight recurring ethical elements entangled in cultural themes observed in the study communities. While some patterns were evident across sites, these reflections are grounded in the lived experiences of participants from these particular population groups and regions and cannot be generalized for all SEA populations.

### Local concepts and interpretation of death

Through community engagement, we observed the value of understanding local concepts around death, interpretations and the coping mechanisms before conducting a large-scale VA study. Participants framed death through diverse spiritual lenses as a release from suffering, a karmic continuation, or divine will. Recognizing these interpretations is essential, and failure to account for such beliefs can lead to mistrust, emotional distress, or refusal to participate (Sreenivas et al., 2023).

Similar to formative research, our qualitative methods allowed us to understand how various cultures interpret death and guide VA implementation in ways that are ethically sound and tailored to the specific context of the community. A study in rural Ethiopia highlighted the importance of local terms and concepts for illnesses and causes of death, and adapted VA questionnaires to ensure cultural relevance and accuracy (Fottrell et al., 2012). As such, this comprehensive approach can help bridge existing knowledge gaps and respects the local concepts around the topic of research rather than instituting pre-existing theoretical models (Brynne, 2019).

Ghanian studies have similarly shown that understanding health beliefs enhances community acceptance and reporting accuracy (Byass et al., 2010). These align with broader calls for addressing epistemic injustice by integrating local knowledge into global health practices (CIOMS, 2016; Fricker, 2007).

### Addressing participants’ emotional distress

Weighing the risks and benefits of research is crucial. Conducting VA research on sensitive topics like death can trigger emotional distress, negative moods, or even depression among participants. In conflict-affected regions like Myanmar, where populations already endure trauma from violence and displacement, such risks are heightened, necessitating careful ethical considerations to avoid retraumatizing participants (Yamada & Matsushima, 2020). Best practices include careful selection of interview timing, location, and conversational procedures prior to interviews to avoid the potential distress and discomfort (Htun et al., 2023).

The study’s findings emphasize the vital role of religion, community support and family networks in coping with grief. Despite diverse beliefs, participants consistently emphasized spiritual and social mechanisms for healing. These insights highlight the need to integrate community engagement into VA research design, ensuring alignment with local practices (Hinga et al., 2021). Ethical guidelines outlined in the CIOMS (Council for International Organizations of Medical Sciences) further mandate upholding participants’ dignity by offering private, supportive environments such as involving family members and training interviewers in empathetic, trauma-sensitive approaches (CIOMS, 2016). Ultimately, combining emotional support with data collection not only improves research quality but also fosters community trust and well-being, bridging scientific and ethical imperatives (Acoba, 2024; Harandi et al., 2017).

### Attending interviewers’ emotional well-being

Research staff frequently exposed to grief and trauma can experience emotional exhaustion, burnout, and moral injury, and the emotional well-being of frontline research workers is often overlooked (Smith, 2021).

Additionally, conducting research in conflict zones could present unique challenges that further heighten their stress levels. Studies suggest that structured debriefing, supportive supervision, and stress management training can protect staff well-being and retention (Dossett et al., 2021; Evans et al., 2023; Nguyen et al., 2021).

Comprehensive ethical training should also be considered during the recruitment process to prepare researchers for the emotional challenges of VA research. This training should include strategies for handling sensitive subjects and recognizing signs of emotional distress. Protecting the mental well-being of VA research teams is crucial, especially as VA becomes more institutionalized in routine national surveys or data collection in LMICs.

### Integrating local moral frameworks in global bioethics

While traditional bioethics often draws on Western principles such as autonomy and justice, our findings highlight the importance of engaging with culturally grounded moral frameworks when implementing VA in SEA. Participants across the study sites emphasized ethical values rooted in relationality, spiritual harmony, and communal obligations such as respecting religious leaders, acknowledging the soul’s journey, and maintaining collective dignity during mourning. In particular, culturally embedded norms like *arr-nar* and *kreng-jai* at the Thai-Myanmar border reflect a relational ethic that prioritizes social harmony and avoiding burdening others, which can shape individuals’ willingness to participate in VA or express emotional distress (Khirikoekkong et al., 2023). Incorporating these local socio-ethical values into VA design and conduct is essential for fostering trust and ensuring culturally sensitive ethical practice.

### Recommendations for ethics integration

Our findings suggest that ethics committees and review boards must play an active role in safeguarding the emotional welfare of both participants and researchers. Study protocols should include mental health risk assessments and mitigation strategies; plan for ongoing emotional monitoring of field teams and specify culturally appropriate engagement and consent processes. These steps are essential for ensuring that ethical review is not a one-time approval, but a dynamic and culturally responsive process throughout the study lifecycle embedded within global health research (Alam et al., 2020; Scott & Kirk, 2015).

### Strengths and limitations

This study employed effective public engagement methods using a combination of body mapping and FGDs to capture the nuanced beliefs and practices surrounding death in diverse communities. Body mapping, which incorporated colors, icons, emojis, and text helped overcome language and literacy barriers, enabling richer expression of culturally specific concepts. The study findings were verified across methods of data collection, for instance, body mapping and FGDs. A key strength lies in the geographic and cultural diversity of participants, representing rural communities across five SEA countries. This facilitated the identification of both shared ethical considerations and context-specific variations in perceptions of death.

We also acknowledge the study limitations. The findings and interpretations may have been influenced by the researchers’ topic guide and methodology, settings, time and desirability bias. Body mapping may have incurred the visualization and expression difficulties arising from the participants’ disparities in artistic skills and interests (Dew, 2018; Vidal et al., 2023). As this study focused on rural settings, the application of findings may be less applicable to urbanized areas where cultural practices differ. Another limitation of this study is the lack of in-depth comparative analysis across study sites. While acknowledging cultural and religious diversity across SEA, we synthesized the findings across sites to highlight shared ethical considerations. Future research with a larger, more stratified sample may allow for a more detailed exploration of cultural and contextual differences that shape perceptions of death and expectations around VA procedures. Moreover, this study provides insights into local perceptions of death and context-specific ethical considerations relevant to the implementation of VA across selected communities in rural SEA. While we identified some common moral frameworks such as communal obligations, spiritual harmony, and relational ethics these findings are specific to the studied regions and reflect the cultural and religious diversity within and between each site. We caution against broad generalizations and advocate for culturally tailored ethical approaches in similar community-based research.

## Conclusion

This study provides valuable insights into the ethical dimensions of conducting VA research in Southeast Asia's culturally diverse and often marginalized communities. By centering the community perceptions of deaths based on the rich cultural and religious traditions, and prioritizing the mental health and well-being of both participants and researchers, this study sets a foundation for ethically robust VA research that respects local beliefs and practices. The knowledge gained from this study not only addresses gaps in context-specific bioethics principles but also offer transferable lessons to conduct future VA research in other LMICs with similar socio-cultural dynamics.
